# The Dilemma for Early-Stage Conjunctival Mucosa-Associated Lymphoid Tissue Lymphoma: To Treat or Not to Treat?

**DOI:** 10.3390/jpm14090927

**Published:** 2024-08-31

**Authors:** Chi-Chun Yang, Chieh-Chih Tsai

**Affiliations:** 1 Department of Ophthalmology, Taipei Veterans General Hospital, Taipei 112, Taiwan; ccyang12@vghtpe.gov.tw; 2 School of Medicine, National Yang Ming Chiao Tung University, Taipei 112, Taiwan

**Keywords:** mucosa-associated lymphoid tissue lymphoma (MALToma), primary conjunctival lymphoma, comparative retrospective study, prognostic factor, MALT international prognostic index (IPI), CD43

## Abstract

Background: Primary ocular adnexal mucosa-associated lymphoid tissue lymphoma (MALToma) is typically treated with radiotherapy. Some studies suggested a “wait and watch” approach due to the adverse effects of radiotherapy. However, the benefits of observation for localized conjunctival MALToma remain unclear. Therefore, we aimed to explore the clinical course of early-stage conjunctival MALToma, distinguish heterogeneity between T1 and T2 patients, and identify prognostic factors. Methods: This retrospective study involved patients with stage T1–T2 conjunctival MALToma and lasted >6 months. Clinical characteristics were compared between T1 and T2 subjects. Prognostic factors were examined with Cox regression. Results: The research comprised 32 subjects with early-stage conjunctival MALToma, of whom 25% underwent observation. No individuals expired regardless of choosing observation or radiotherapy. The T1 patients were younger (*p* = 0.002) and more inclined towards observation only (*p* = 0.035) than the T2 subjects. Despite more of the T1 patients undergoing watchful waiting than the T2 subjects, the T1 patients seemed to have longer systemic relapse-free survival than the T2 subjects (17 vs. 13 years, *p* = 0.343). CD43 may imply poor prognosis (*p* = 0.049). Conclusions: Careful observation may be suggested for early-stage conjunctival MALToma. While more of the T1 individuals were younger and chose observation than the T2 patients, survival seemed longer in the T1 subjects without significance. CD43 may indicate shorter survival in early-stage cases.

## 1. Introduction

Conjunctival lymphoma is the third most frequently occurring primary malignancy of the ocular surface and has become increasingly common in recent years [1,2,3]. Of them, primary conjunctiva extranodal marginal zone lymphoma (EMZL), also known as mucosa-associated lymphoid tissue (MALT) lymphoma (MALToma), is the most common subtype of conjunctival lymphoma, which accounts for approximately 80% of conjunctival B-cell non-Hodgkin’s lymphomas (NHLs) [4,5]. Primary conjunctival MALToma typically manifests as a chronic subepithelial salmon pink-like conjunctival patch, approximately 10–33% of which have concurrent systemic involvement [4,5,6]. Though the pathophysiology of conjunctiva MALToma remains elusive, several treatment modalities have been proposed, which include radiotherapy, cryotherapy, intralesional immunotherapy (rituximab/interferon), oral antibiotic trial, chemotherapy, and a combination of radiation and chemotherapy [5,7].

Additionally, considering most ocular adnexal MALTomas present as isolated lesions with an indolent course, observation had been suggested for primary cases [8,9]. However, most relevant studies included patients of various stages or mixed diagnoses (e.g., follicular lymphoma and diffuse large B-cell lymphoma) without explicitly focusing on early-stage conjunctival MALToma [8,9]. For example, one article indicated watchful waiting as an acceptable treatment option for asymptomatic subjects based on retrospectively reviewing 75 patients with primary ocular adnexal MALToma across a wide range of stages ranging from Ann Arbor stage I to IV [8]. Currently, there is a lack of evidence specifically for early-stage cases, which highlights the potential and value of our study.

However, the existing body of literature on primary ocular adnexal MALToma was mainly centered around the comparative analysis of various treatment modalities for patients exhibiting a diverse spectrum of tumoral involvement, which ranged from conjunctival-only to more widespread conditions involving the orbit with or without the conjunctiva [8,10,11,12,13]. There was a dearth of research that focused on the disparities in clinical parameters and outcomes between isolated conjunctival MALToma (stage T1) and conjunctival MALToma with orbit/lacrimal gland involvement (stage T2). In previous studies, these two types of MALTomas were typically classified together as the primary ocular adnexal MALToma of Ann Arbor stage IE [8,11,13,14,15]. 

In light of this, further research is necessary to gain a better understanding of the differences between these two types of conjunctival MALTomas to optimize the current management for affected patients. Though radiotherapy is widely regarded as the standard treatment for localized primary ocular adnexal MALToma due to its high efficacy in local control, considering its potential adverse effects, such as cataracts, retinitis, or secondary lymphoma, it would be highly advantageous to identify the subset of Ann Arbor stage IE patients who carry a higher risk of local or systemic relapse [14,16,17]. This identification would enable us to provide further intervention to those who may truly benefit from it and increase our vigilance during follow-up examinations. 

In addition, most proposed studies investigated clinical factors that could impact the outcomes in patients with the ocular adnexal MALToma of mixed stages ranging from stage T1 to T4, which warrants further research specifically targeting prognostic factors for early-stage conjunctiva MALToma confined only to the conjunctiva to improve the care and follow-up program for individuals with localized conjunctival MALToma [2,10,11,12].

Therefore, the purpose of our study is to explore the heterogeneity between primary isolated conjunctival MALToma (stage T1) and primary conjunctival MALToma with orbit/lacrimal gland involvement (stage T2) in terms of clinical characteristics and outcomes at long-term follow-up. In addition, we aim to identify prognostic factors affecting survival outcomes exclusively for early-stage conjunctival MALToma.

## 2. Materials and Methods

### 2.1. Patients

This retrospective study reviewed the medical records of 32 patients diagnosed with primary localized conjunctival MALToma and who underwent follow-ups at the first three medical centers in Taiwan from 2005 to 2024. This study was approved by the Ethical Review Committee of Taipei Veterans General Hospital, Taiwan. All procedures adhered to the tenets of the Declaration of Helsinki for research involving human subjects. As all patients were de-identified from our datasets, the necessity for informed consent was waived. We included patients (a) with pathologically confirmed conjunctival MALToma without systemic involvement or history of lymphoma/leukemia, (b) receiving complete staging workup including blood tests and positron emission tomography/computed tomography (PET/CT) or magnetic resonance imaging (MRI), (c) who have been followed up for more than 6 months since the excisional biopsy date. We excluded patients with localized ocular adnexal MALToma without conjunctival involvement, incomplete medical charts, initial systemic dissemination, recurrent/relapsed disease status, or who were undergoing relevant treatments elsewhere. 

Each included patient received an excisional biopsy by the single oculoplastic specialist (C.C. Tsai) with the extent of removal being as large as possible without decreasing the function and appearance of the affected eyes. The specimen was examined by a pathology specialist with an immunohistochemistry stain to confirm the diagnosis according to the WHO guidelines. CD43 was checked using immunohistochemical stains in all specimen sections. This method was implemented during the retrospective period. Clonal comparison using flow cytometry was not performed.

The treatment options, including observation with close follow-up, radiotherapy, intralesional interferon, chemotherapy, and target therapy, were provided to patients, and the shared-decision making was conducted to achieve the final plan. Each patient had a returning visit at Ophthalmology and Radiology/Hematology OPD, scheduled every 3 months, with an annual MRI/CT follow-up after the treatment course. We employed two staging systems, the Ann Arbor Staging System and the American Joint Committee on Cancer (AJCC) TNM Staging System, to determine the stage of all patients [5]. In the event of bilateral involvement, we classified the condition as Ann Arbor stage I. The MALT international prognostic index (IPI) was also calculated as a possible prognostic indicator for each patient [18].

### 2.2. Definition and Outcome Measures

In our research, patients who met the criteria for inclusion were further divided into two groups: primary isolated conjunctival MALToma (group 1) and primary conjunctival MALToma with extension to the orbit or lacrimal gland (group 2). These groups corresponded to the T1N0M0 and T2N0M0 stages of AJCC, respectively. Treatment response was evaluated 6 months after initial treatment with consultation with a hematologist/radio-oncologist. Complete response (CR) was determined as no clinically evident tumor requiring further interventions, as evidenced by clinical inspection and CT/MRI scans. Relapse-free survival (RFS) was defined as the time interval between the diagnosis date and the occurrence of distant or local relapse, mortality resulting from any cause, or the last follow-up, whichever occurred first. Overall survival (OS) was defined as the time from diagnosis to death. For patients who survive the entire follow-up period, their survival data are censored at the last recorded assessment date.

### 2.3. Statistical Analysis

The normality of the data was checked by using the Shapiro–Wilk test. For continuous variables with normal distribution, we presented the mean ± standard deviation, while for non-normally distributed data, we presented medians (interquartile range [IQR], P25–P75). We summarized categorical variables as the number of cases and percentages (%). The distribution of demographics and clinical parameters between the two groups was compared using a chi-square test for categorical variables and the Student’s *t*-test or the Mann–Whitney U test for continuous variables. 

We conducted a survival analysis using the Kaplan–Meier method and compared the survival curves with a log-rank test. *p* value < 0.05 was considered statistically significant. The study utilized Cox proportional-hazards regression to examine various parameters that may act as risk factors for RFS. The output of Cox regression statistics consists of hazard ratio (HR) with 95% confidence intervals (95% CIs). Multivariate Cox regression analysis with backward elimination was carried out for variable selection for a more efficient and conservative selection process. The backward elimination starts with a comprehensive set of predictors and subsequently eliminates those that do not yield significant contributions. All analyses were performed using the SPSS ver. 23.0 (IBM Corp., Armonk, NY, USA).

## 3. Results

### 3.1. Patient Characteristics

Overall, 34 patients were diagnosed with primary conjunctival MALToma over the 19-year period, while 2 of them were excluded due to systemic involvement at initial presentation. The analysis included only patients with localized conjunctival MALToma, with or without lacrimal gland/orbit involvement (AJCC stage T1–T2). According to Table 1, the median age of 32 subjects was 45 years with 63% being female. In addition, 81.2% of the participants achieved a MALT-IPI index score of 0, indicating low risk. The remaining 18.8% fell into the intermediate-risk category, with none classified as high risk.

Of the included patients, 8 (25.0%) chose watchful waiting, 17 (53.1%) received radiotherapy, and 6 (18.8%) opted for chemotherapy. Over a median follow-up period of 6 years, 3 out of 32 patients (9.4%) experienced distant relapse, with no reported fatalities. Of these, one patient chose initial observation and subsequently developed systemic relapse with splenic and multiple lymphadenopathies 4 years later. The remaining two patients, who initially opted for chemotherapy, experienced gastrointestinal relapse 6 and 11 years later, respectively. The average relapse-free survival duration for this cohort of 32 cases was 15.3 years.

### 3.2. Subgroup Analysis

The remaining 32 patients were further divided into two groups and included in the analysis: primary isolated conjunctival MALToma (T1, 22 patients) and primary conjunctival MALToma with extension to orbit/lacrimal gland (T2, 10 patients). 

As shown in Table 2, the average age was 40 (IQR, 30–48) with 15 female patients (68%) in group 1, while patients in group 2 were averagely aged 58 (IQR, 50–74) with 5 female subjects (50%). Both groups were diagnosed with Ann Arbor stage IE and had no relevant history of lymphoma or leukemia. The demographics, underlying diseases, and treatment outcomes of the two groups were compared. 

The T1 patients with more limited tumor distribution exhibited a statistically significantly younger age (*p* = 0.002) and a lower probability of suffering from hypertension (one patient, 4.5% vs. five patients, 50%, *p* = 0.006) compared to the T2 patients. There were no significant differences between the two groups in gender, laterality, duration of symptoms, underlying diseases, malignancy history, cigarette smoking, MALT IPI score, and positivity of CD43. There appeared to be an inclination towards female representation in group 1, with a proportion of females twice that of males, compared to group 2, which had an equal distribution of both genders. However, the difference was not statistically significant.

### 3.3. Treatment and Outcomes

Each patient was provided with a range of treatment options, such as observation, radiotherapy, chemotherapy, or immunomodulatory therapy, and underwent comprehensive discussions with a qualified radio-oncologist or hematologist. Table 3 compared the interventions and prognosis between two arms. The proportion of watchful waiting without further interventions was significantly higher in group 1 compared to group 2 (eight patients, 25% vs. zero patients, 0%, *p* = 0.035). Otherwise, there was no significant difference regarding the distribution of other interventions among the two groups. 

**Figure 1:**
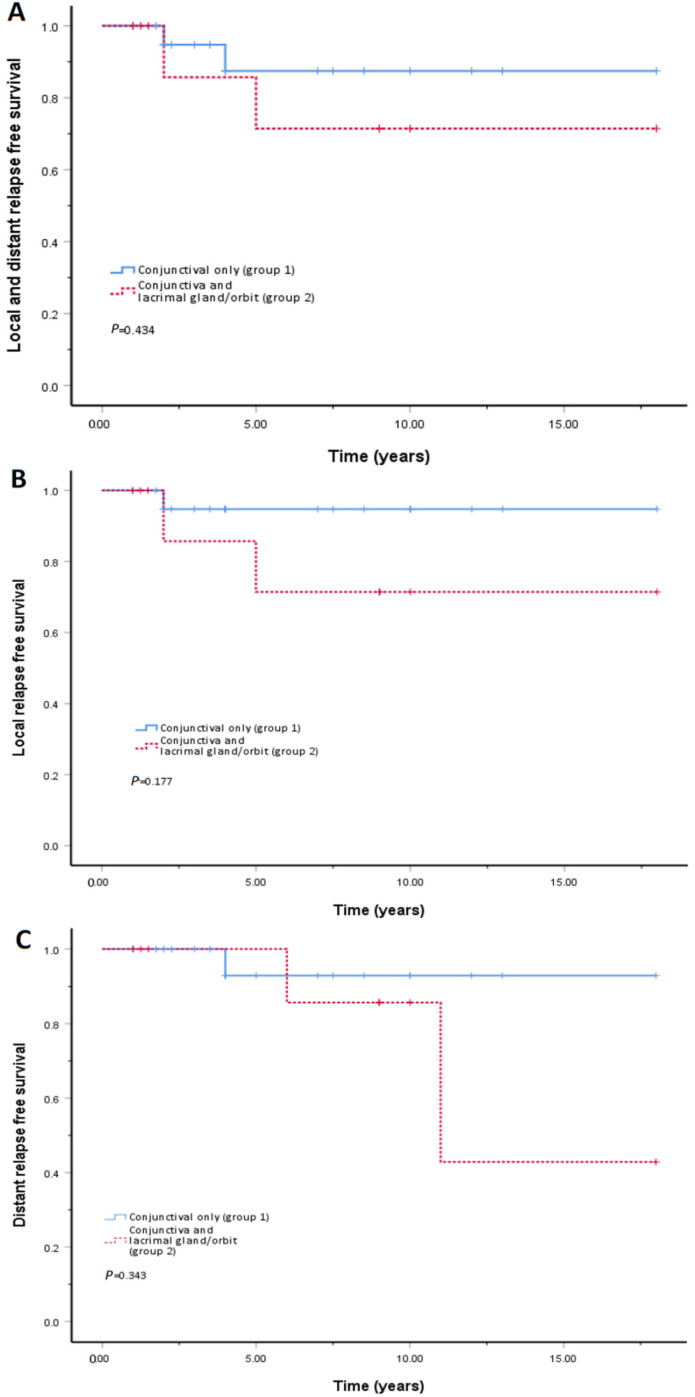
Comparing the Kaplan–Meier survival curve between primary isolated conjunctival MALToma (T1, group 1) and primary conjunctival MALToma with lacrimal gland/orbit involvement (T2, group 2). (**A**) Local and distant relapse-free survival; (**B**) Local relapse-free survival; (**C**) Distant relapse-free survival.

The median follow-up period was 4 (IQR, 2–10) and 9 years (IQR, 1.5–10) in group 1 and group 2 (*p* = 0.305), respectively. Both groups demonstrated CR after the initial treatment, whereas patients who opted for observation showed stable disease. No difference in local or distant relapse rate was detected between the two groups. However, there was a tendency for more patients to experience relapse in group 2, compared to group 1, despite no statistical significance. Figure 1 displayed the survival curve of the two groups. The 5-year RFS rate was 87.4% and 71.4% in group 1 and group 2, respectively. There was an average of 16.1 years (95 CI, 13.7–18.6) before T1 patients experienced local or distant relapse in comparison to 13.9 years (95 CI, 9.0–18.7) for T2 patients without significance (*p* = 0.434). No patient died throughout the entire follow-up period. The overall survival (OS) rate was 100% in both groups.

### 3.4. Influencing Factors for Prognosis

Table 4 provided a summary of the Cox proportional analysis for patients with primary conjunctival MALToma at T1 and T2 stages. No variable had a significant impact on RFS in univariate analysis. Multivariate analysis with stepwise selection revealed the presence of CD43 (*p* = 0.049) as an independent factor associated with shorter RFS.

Kaplan–Meier curves of relapse-free survival for all included patients diagnosed as primary conjunctival mucosa-associated lymphoid tissue lymphoma (MALToma) with and without other ocular adnexal extension.

## 4. Discussion

This research involved 32 participants with localized conjunctival MALToma (Ann Arbor stage IE) and did a subgroup analysis based on the AJCC stages to compare the characteristics and outcomes between isolated conjunctival MALToma (AJCC stageT1) and conjunctival MALToma with extension to other ocular adnexa (AJCC stage T2). To the best of our knowledge, this is the first study to stratify patients with conjunctival MALToma of Ann Arbor stage IE into two distinct groups and investigate the heterogeneity between them. 

In our cohort of 32 patients, eight individuals opted for observation without additional interventions. Among them, one patient experienced a distant relapse 4 years later but achieved disease stability after chemotherapy. No patients died or declined in daily functionality. Although prior studies had proposed that watchful waiting could be an option for primary ocular MALToma, there was a lack of evidence exclusively targeting Ann Arbor stage IE conjunctival MALToma. For instance, one retrospective study indicated no difference in time to start new treatments between the observation and radiotherapy groups by analyzing 75 patients with ocular adnexal MALToma of Ann Arbor stage IE–IV [8]. This highlighted the importance and potential of our study in providing data regarding the clinical course of early-stage conjunctival MALToma.

In our analysis, we observed that patients in group 1 were younger and less likely to have hypertension compared to those in group 2. Older age had been indicated as a predicting factor for unfavorable outcomes in several studies on ocular adnexal MALToma [5,10,11,12,19,20]. In a retrospective study, univariate logistic regression analysis revealed that advanced age was linked to a higher likelihood of systemic involvement at the time of initial presentation (OR = 1.05, *p* = 0.012), as well as a reduced probability of achieving a complete response following treatment (OR = 0.95, *p* = 0.009) among patients with primary ocular adnexal MALToma [10]. Additionally, older age also served as one of the poor prognostic indicators in the IPI index score, which was detected to be a significant factor that was able to isolate patients with higher risks of treatment failure [12]. Accordingly, our finding that subjects in group 1, who were defined to have less conjunctival MALToma involvement compared to those in group 2, presented at a younger age may align with previous research suggesting younger age as a favorable prognostic indicator [10,11,12,19]. 

Our analysis also revealed that hypertension was more prevalent in patients with primary MALT lymphoma affecting the conjunctiva and orbit/lacrimal gland (group 2) compared to patients with MALT lymphoma localized exclusively in the conjunctiva (group 1). Since these results were obtained through univariate analysis, the higher prevalence of hypertension in T2 subjects was likely associated with their more advanced age. Furthermore, one case series utilizing immunohistochemical analyses found the expression of prorenin receptor and angiotensin II type 1 receptor (AT1R) in surgically removed conjunctiva MALToma tissue. The study suggested that activations of the tissue renin-angiotensin system (RAS) are associated with the pathogenesis of conjunctival MALToma by stimulating subsequent events involving extracellular matrix turnover and tumor angiogenesis [21]. Additionally, the prorenin receptor has been reported to contribute to the pathogenesis of hypertension [21,22]. Taken together, the common pathogenic pathway shared by conjunctival MALToma and hypertension may partially account for the higher prevalence of hypertension occurring in patients with a more advanced involvement of MALToma. 

Additionally, there was a tendency for a higher proportion of female participants and bilaterality in group 1 compared to group 2 without significance. A retrospective cohort study involving 182 patients with primary ocular adnexal MALToma indicated a higher prevalence of female patients in cases with conjunctival presentation, with a female-to-male ratio of 2:1. This trend was not observed in cases where MALToma was located in the orbit or lacrimal gland [11]. Another retrospective study involving 198 patients also compared clinical features and prognosis between primary conjunctival MALToma and other ocular adnexal MALToma treated from 1995 to 2015 in Korea [10]. In line with our study, they also identified that patients with conjunctival MALToma were younger, primarily female, and exhibited a higher rate of bilaterality than other ocular adnexal EMZLs [10]. However, the two studies mentioned above included ocular adnexal MALToma from localized to the conjunctiva (stage T1) to systemic involvement (stage T4), which differ from the current study exclusively focusing on localized primary conjunctival MALToma with or without orbit/lacrimal gland involvement (AJCC stage T1 to T2). Therefore, the value of our study was providing clinical information and outcomes specifically for very early-stage conjunctival MALToma, which was mostly grouped together with conjunctival MALToma of stage T4 in earlier research.

Several studies had investigated influencing factors for survival outcome in primary ocular adnexal MALToma [2,10,11,12,23]. Nonetheless, there was a lack of studies exploring prognostic factors exclusively for primary conjunctival MALToma at an early stage. Therefore, Cox proportional regression was performed to provide relevant information. Multivariate analysis showed that CD43 was associated with shorter RFS (*p* = 0.049), while higher MALT IPI scores appeared to negatively impact RFS with borderline significance (*p* = 0.082). This finding aligned with the observations from a retrospective study conducted among 20 patients with ocular adnexal MALToma at Ann Arbor stage I–IV. According to their survey, CD43 positivity was associated with a 14 times higher likelihood of failure-free survival (*p* = 0.035) [15]. This may be explained by the fact that CD43 expression is rarely detected on non-neoplastic B-cells, which renders it an immunophenotypic marker suggestive of malignancy [15,24]. 

The present study found that there was no significant association between watchful waiting and reduced progression-free survival. This finding was compatible with one retrospective article from the Kyoto Clinical Hematology Study Group, which included 75 patients with primary ocular adnexal MALToma, with 92% of them at Ann Arbor stage IE [8]. The study found that the time to initiate new treatment did not differ significantly between the watchful waiting and radiotherapy groups [8]. Consequently, they suggested that watchful waiting may be considered a viable treatment option for ocular adnexal MALToma, particularly in the case of asymptomatic patients [8].

This study was subject to several limitations. First, due to its retrospective nature, the non-standardized treatment plan, and follow-up schedule, caution should be exercised when interpreting and comparing characteristics between the two groups. Second, the possibility of selection bias should be acknowledged, as the study was conducted at a tertiary medical center. Third, the small sample size potentially diminished statistical power. However, to the best of our knowledge, this study is the first to compare clinical parameters and courses in early-stage primary conjunctival MALToma between stage T1 and T2.

## 5. Conclusions

The present study exclusively included patients with primary conjunctival MALToma at stage T1 and T2, followed by a comparative analysis between the two groups, which had not been proposed in prior studies. The results revealed that patients with primary MALToma confined to the conjunctiva (T1 stage) were significantly younger and more inclined to receive observation than those with a concurrent involvement of the ocular adnexa (T2 stage). Despite this, the T1 individuals seemed to have longer RFS than the T2 patients without significance. The findings suggested that watchful waiting may be a feasible treatment option for T1 patients. Additionally, CD43 may be a poor prognostic factor for early-stage conjunctival MALToma.

## Figures and Tables

**Table 1:** Clinical characteristics and outcomes of early-stage primary conjunctival MALToma (T1 and T2).

Demographics	Total (32 Patients)	Outcomes	Total (32 Patients)
Age (y)	45 (35–59)	Treatment	
Gender (male:female)	12:20	Watchful waiting	8 (25.0%)
Unilateral:bilateral	15:17	Radiation therapy	17 (53.1%)
Duration of symptoms (month)	3 (3–8)	Chemotherapy	6 (18.8%)
Underlying diseases		Target therapy	2 (6.3%)
Diabetes mellitus	4 (12.5%)	Intralesional therapy	2 (6.3%)
Hypertension	6 (18.8%)	Overall follow-up (year)	6 (2–10)
Previous tumor	4 (12.5%)	Outcome	
Previous lymphoma/leukemia	0 (0.0%)	Local relapse	3 (9.4%)
Autoimmune disease	1 (3.1%)	Distant relapse	3 (9.4%)
Hepatitis B	6 (18.8%)	Death of any causes	0 (0.0%)
Hepatitis C	0 (0.0%)	Relapse-free survival (RFS)	
H. pylori infection	1 (3.1%)	Local or distant RFS (year)	15.3 (12.8–17.7)
Personal and medication history		Local RFS (year)	16.0 (13.8–18.1)
Immunosuppressive drugs	1 (3.1%)	Distant RFS (year)	15.3 (12.6–18.1)
Smoking	2 (6.3%)		
LDH (U/L)	177 (144–201)		
Beta2M (ng/mL)	1275 (1151–1705)		
Ann Arbor stage	IE		
AJCC stage	T1 + T2		
CD43	8 (25.0%)		
MALT-IPI score			
Low risk	26 (81.2%)		
Intermediate risk	6 (18.8%)		
High risk	0 (0.0%)		

MALToma (mucosa-assisted lymphoid tissue lymphoma), AJCC (American Joint Committee on Cancer), LDH (lactate dehydrogenase), Beta2M (beta 2 microglobulin), MALT-IPI (MALT lymphoma international prognostic index). The continuous variables are presented as median (P25–P75) and analyzed by the Mann–Whitney U test. The categorical variables are shown as numbers (proportion) and analyzed using Fisher’s exact and chi-square tests. The RFS is displayed as mean (95% confidence interval) and analyzed with the Kaplan–Meier method.

**Table 2:** Comparison of clinical characteristics between primary isolated conjunctival MALToma (T1) and primary conjunctival MALToma with lacrimal gland/orbit involvement (T2).

	Conjunctival-Only (T1, 22 Patients)	Conjunctiva and Lacrimal Gland/Orbit (T2, 10 Patients)	*p* Value (T1 vs. T2)
Age (y)	40 (30–48)	58 (50–74)	**0.002**
Gender (male:female)	7:15	5:5	0.438
Unilateral:bilateral	9:13	6:4	0.267
Duration of symptoms (month)	3 (1–3)	8 (3–12)	0.919
Underlying diseases			
Diabetes mellitus	1 (4.5%)	3 (30.0%)	0.079
Hypertension	1 (4.5%)	5 (50.0%)	**0.006**
Previous tumor	2 (9.1%)	2 (20.0%)	0.572
Previous lymphoma/leukemia	0 (0.0%)	0 (0.0%)	NA
Autoimmune disease	1 (4.5%)	0 (0.0%)	1.000
Hepatitis B	5 (22.7%)	1 (10.0%)	0.637
Hepatitis C	0 (0.0%)	0 (0.0%)	NA
H. pylori infection	1 (4.5%)	0 (0.0%)	1.000
Personal and medication history			
Immunosuppressive drugs	1 (4.5%)	0 (0.0%)	1.000
Smoking	0 (0.0%)	2 (20.0%)	0.091
Ann Arbor stage	IE	IE	NA
AJCC stage	T1	T2	NA
LDH (U/L)	182 (151–202)	170 (141–198)	0.493
Beta2M (ng/mL)	1195 (1122–1457)	1678 (1386–1862)	0.674
CD43	5 (22.7%)	3 (30.0%)	0.681
MALT-IPI score			0.060
Low risk	20 (90.9%)	6 (60%)	
Intermediate risk	2 (9.1%)	4 (40%)	
High risk	0 (0.0%)	0 (0.0%)	

MALToma (mucosa-assisted lymphoid tissue lymphoma), AJCC (American Joint Committee on Cancer), LDH (lactate dehydrogenase), Beta2M (beta 2 microglobulin), MALT-IPI (MALT lymphoma international prognostic index), NA (not available). The continuous variables are presented as median (P25–P75) and analyzed by the Mann–Whitney U test. The categorical variables are shown as numbers (proportion) and analyzed using Fisher’s exact and chi-square tests. The bold figures indicate a *p* value less than 0.05.

**Table 3:** Comparison of treatment and outcomes between primary isolated conjunctival MALToma (stage T1, group 1) and primary conjunctival MALToma with other ocular adnexal involvement (stage T2, group 2).

	Conjunctival-Only (T1, 22 Patients)	Conjunctiva and Lacrimal Gland/Orbit (T2, 10 Patients)	*p* Value
Treatment			
Watchful waiting	8 (25.0%)	0 (0.0%)	0.035
Radiation therapy	10 (45.5)	7 (70.0%)	0.265
Chemotherapy	3 (13.6%)	3 (30.0%)	0.346
Target therapy	1 (4.5%)	1 (10.0%)	0.534
Intralesional therapy	2 (9.1%)	0 (0.0%)	1.000
Overall follow-up (year)	4 (2–10)	9 (1.5–10)	0.305
Outcome			
Local relapse	1 (4.5%)	2 (20.0%)	0.224
Distant relapse	2 (9.1%)	2 (20.0%)	0.572
Death of any causes	0 (0.0%)	0 (0.0%)	NA
Local and distant RFS (year)	16.1 (13.7–18.6)	13.9 (9.0–18.7)	0.434
Local RFS (year)	17.2 (15.6–18.8)	13.9 (9.0–18.7)	0.177
Distant RFS (year)	17.0 (15.1–18.9)	13.3 (8.6–18.0)	0.343

MALToma (mucosa-assisted lymphoid tissue lymphoma), relapse-free survival (RFS), NA (not available). The non-normally distributed continuous variables are exhibited as median (interquartile range, P25–P75) and analyzed by the Mann–Whitney U test. The categorical variables are shown as numbers (proportion) and analyzed using Fisher’s exact and chi-square tests. The RFS is displayed as mean (95% confidence interval) and analyzed with the Kaplan–Meier method. A *p* value less than 0.05 is considered significant.

**Table 4:** Univariate and multivariate Cox proportional hazard regression analysis of the relapse-free survival for all included patients with primary conjunctival MALToma (stage T1 and T2).

	Univariate			Multivariate		
	HR	95% CI	*p*	HR	95% CI	*p*
Age	1.019	0.960–1.082	0.538			
Male (vs. female)	0.643	0.067–6.188	0.702	0.165	0.007–3.812	0.261
Bilateral (vs. unilateral)	2.208	0.230–21.23	0.493			
T2 stage (vs. T1 stage)	2.152	0.297–15.59	0.448	4.029	0.453–35.86	0.212
MALT-IPI score = 1 (vs. 0)	4.484	0.464–43.34	0.195	19.77	0.682–573.7	0.082
CD43	4.342	0.601–31.37	0.146	12.64	1.016–157.4	0.049
Watchful waiting	1.087	0.113–10.47	0.943			

MALToma (mucosa-assisted lymphoid tissue lymphoma), HR (hazard ratio), CI (confidence interval), and MALT-IPI (MALT lymphoma international prognostic index). A *p* value less than 0.05 is considered significant.

## Data Availability

Data are contained within the article.
